# Languagelike-Specificity of Event-Related Potentials From a Minimalist Program Perspective

**DOI:** 10.3389/fpsyg.2019.02828

**Published:** 2019-12-13

**Authors:** Daniel Gallagher

**Affiliations:** Department of Linguistics and Literature, Graduate School of Humanities, Kyushu University, Fukuoka, Japan

**Keywords:** minimalist program, faculty of language, event-related potentials, math, music

## Abstract

In this mini-review, I use event-related potential (ERP) studies to test the minimalist program (MP) prediction that organisms with the faculty of language cognitively process languagelike systems in a qualitatively distinct manner. I first discuss “languagelike” as a technical term defined by recursion criteria. From this definition and using a generative perspective, I show that certain domains of math and music can be considered languagelike. These domains are then used as case studies to test whether or not different languagelike systems are cognitively processed in a similar manner. This is done by investigating the elicitation of common language-related ERPs (namely, the left-anterior negativity (LAN), N400, and P600) in these languagelike systems. I show that these systems do indeed elicit the same language-related ERPs, supporting the claim that different languagelike systems are processed similarly. I then discuss discrepancies between these systems, as exemplified by the P3, and I provide plausible accounts for interpreting those results. I ultimately conclude that present data on the LAN, N400, and P600 disprove language-specificity but that languagelike-specificity remains plausible, and as yet there is no reason to reject MP’s prediction that languagelike systems are processed in a qualitatively distinct way.

## Introduction

As a research program with its foundation in the biolinguistics framework, the Minimalist Program (MP) “seeks the simplest formulation of Universal Grammar (UG),” which is “the theory of the biological endowment of the relevant components of the faculty of language (FL)” (Chomsky, 1995, p. viii). Thus, though each exists as its own object of inquiry, any prediction of FL is a prediction of UG, which is a prediction of MP (though not necessarily vice versa).

Hauser et al. note that “investigations of [FL] should include domains other than communication” (Hauser et al., 2002, p. 1571). In this mini-review, I use functional neuroimaging studies to test the MP prediction that there is “a qualitative difference in the way in which organisms with [FL] approach and deal with systems that are languagelike and others that are not” (Chomsky, 1965, p. 56)^1^.

To that end, I first precisely define what constitutes a languagelike system and establish some testable systems that meet this definition. I then test for specificity to languagelike systems (“languagelike-specificity”) by reviewing event-related potential (ERP) studies and demonstrating similarities of cognitive processes (“cognitive overlap”) across different languagelike systems.

It should be said at the outset that it is not possible to comprehensively cover the abundance of language-related ERP studies within this mini-review. I have therefore carefully selected only those studies most relevant to the discussion herein, and I recommend that the interested reader check Kutas and Federmeier (2011), Brouwer and Crocker (2017), and Nieuwland (2019) for comprehensive reviews on language-related ERPs.

## Languagelike Systems

According to Chomsky et al., there are two empirical, non-negotiable characteristics of language: discrete infinity and displacement (Chomsky et al., 2017, p. 3). Discrete infinity refers to the infinite generative capacity of grammatical sentences from a finite set of symbols, or the ability to “make infinite use of finite means” (Chomsky, 1965, p. 8). Displacement refers to the maintaining of a noun phrase’s thematic relation to a verb, while displacing it from its base position, such as is found in active/passive voice alternation (Chomsky et al., 2017, p. 3). Note, however, that these two characteristics are simply the consequence of an underlying computational mechanism. We call this mechanism Merge, a fundamental set-formation operation that produces a new syntactic object *K* from two syntactic objects X and Y, such that *K* = {X,Y}. Importantly, it is the *recursive* application of Merge that is considered sufficient to account for both discrete infinity and displacement (Chomsky et al., 2017, pp. 3–4). Thus, FL is characterized by recursion^2^, which is often regarded as the most fundamental feature of language and consequently gives us a suitable working definition: a languagelike system is one that utilizes recursion.

### Recursion

To move further, we must understand recursion. It is tempting to equate recursion with embedding, for example in the use of recursive possessives (as in “my father’s father’s father’s...”), in the use of recursive relative clauses (as in “the boy that wore the shirt that got dirty at the game that...”), and so on. However, this oversimplification is a mischaracterization that has led to confusion over whether or not recursion exists in all human language (see Everett, 2005 and Nevins et al., 2009, for the famous debate on Pirãha exceptionality). In fact, embedding is a property and evidence of recursion, but recursion is not limited to embedding. Watumull et al. formally describe recursion by three criterial properties: computability, definition by induction, and mathematical induction (Watumull et al., 2014, p. 1). Computability refers to output being generated deterministically by conditional branching, as in a Turing machine: “IF in state *q_i_* reading symbol *x_i_* on the tape, THEN write *y_i_*, move one space, transition to state *q_j_*” (Watumull et al., 2014, pp. 1–2). A function is computable if its deterministic rules are finitely specified. Definition by induction allows strong generation of increasingly complex structures through stepwise computation (Watumull et al., 2014, p. 2). Lastly, mathematical induction results in an unbounded (i.e., infinite) computable generation of structured expressions. An important distinction is that *generation* can be infinite while *production* is finite due to some arbitrary constraint (Watumull et al., 2014, p. 3). In a Turing machine, such a constraint might be tape length, while in human language, it could be memory limitations, lack of cultural utility (e.g., counting above a certain number), etc. In summary, recursion requires that three criteria are met: (1) computability gives finitely specified rules, (2) definition by induction allows stepwise computation, and (3) mathematical induction provides infinite generative capacity.

### Math and Music

With this definition of a languagelike system, let us take arithmetic sequences and musical prolongation as two case studies. First, consider the famous Fibonacci sequence, defined *F_n_* = *F_n_*_−1_ + *F_n_*_−2_ for each *n* ∈ **N**, with *F*_0_ = 0 and *F*_1_ = 1. This yields {0,1,1,2,3,5,8,13…}, *generating* integers infinitely, and without arbitrary constraints (e.g., “for n < 10”), it will also *produce* integers infinitely. Thus, the three conditions are satisfied: computability is achieved by the finitely specified formula *F_n_*, definition by induction is satisfied by stepwise computation of the formula, and the sequence is generatively unbounded, satisfying mathematical induction. This same procedure can be used to show any arithmetic sequence to be recursive.

Superficially, music and language have many similarities (expressive communication, cultural significance, local variation, etc.), but it is not straightforward whether music is a languagelike system. In their book *A Generative Theory of Tonal Music* (GTTM), Lerdahl and Jackendoff developed a formal grammar for music. The authors first note that a generative theory of music is “a formal description of the musical intuitions of a listener who is experienced in a musical idiom” (Lerdahl and Jackendoff, 1983, p. 1). Let us consider musical prolongation. In music theory, the highest hierarchical level is the tonic (i.e., the resolving pitch) of the key (e.g., in the key C Major, the tonic is C). The tonic is said to *prolongate,* governing all parts of the piece played in relation to it (Lerdahl and Jackendoff, 1983, p. 179). Consider ending “Mary had a Little Lamb” on “it’s fleece was white as.” The omission of “snow” leaves the piece melodically unresolved, illustrating that note’s function as the prolongational head.

Regarding prolongation, GTTM provides four “prolongation reduction well-formedness rules” (PRWFR). Though I will only use the first rule, I include all four (greatly simplified) both for reference and to adequately demonstrate that prolongation satisfies languagelike criteria:

**PRWFR 1:** Every (section of a) piece has a single prolongational head.
**PRWFR 2:** A pitch event *e_i_* can be a direct elaboration^3^ of event *e_j_* in the following ways:
  1. *e_i_* is a strong prolongation of *e_j_* if its notes are identical;
  2. *e_i_* is a weak prolongation of *e_j_* if the roots are identical but some notes differ;
  3. *e_i_* is a progression to or from *e_j_* if the roots differ.
**PRWFR 3:** Every event is either the prolongational head or a recursive elaboration of it.
**PRWFR 4:** (No Crossing Branches) If *e_i_* is a direct elaboration of *e_j_*, every event between them, must be a direct elaboration of *e_i_*, *e_j_*, or some event between them (Lerdahl and Jackendoff, 1983, pp. 214–215).

Here, we have finitely specified generative rules (computability), applied through stepwise computation (definition by induction), and with infinite generative capacity through recursive elaboration (mathematical induction). Thus, musical prolongation satisfies recursion criteria and is indeed a languagelike system.

Note, not all musical structures are languagelike, just as not all mathematical disciplines are, just as not all vocal utterances are. However, this does not preclude their use as empirical tests for languagelike-specificity, since we only consider languagelike subsets of each domain.

## Event-Related Potentials and Languagelike-Specificity

Event-related potentials (ERPs) are stimulus-induced, time-locked, averaged electric potentials in the brain measured by electroencephalography (EEG). EEG is a common neurolinguistic research method with high temporal resolution, well suited for studying the time-course of language processing (Stemmer and Rodden, 2015, pp. 477–478; see also Burle et al., 2015, for limitations). Table 1 summarizes all ERPs reviewed henceforth.

**Table 1:** A simplified summary of ERPs elicited by violations of language and languagelike systems.

Domain	Violation type	Violation example	ERPs	Source
Language	Semantics	“The cat will bake.”	N400	Federmeier et al. (2002), Osterhout et al. (2004), Lau et al. (2008)
Language	Syntax	“The blouse was on ironed.”	P600	Osterhout and Holcomb (1992), Kutas et al. (2006), Brouwer and Crocker (2017)
Language	Morphosyntax	“The clerk were severely underpaid.”	LAN, P600	Barber and Carreiras (2005), Molinaro et al. (2011)
Math	Arithmetic sequence	“7 10 13 16 19 22 24”	LAN, P600	Núñez-Peña and Honrubia-Serrano (2004)
Math	Arithmetic operation	“7 × 4… 24”	N400, P3	Niedeggen et al. (1999)
Music	Prolongation	[out-of-key chord]	RATN, P600	Besson and Faïta (1995); Patel et al. (1998)
Music	Meter	[deviant accent]; [empty beat]	MMN, P3	James et al. (2012), Bouwer et al. (2014)

*In each example provided, words/numbers were presented visually one at a time, except for musical stimuli, which were presented aurally. Underlined words/numbers represent the critical stimulus, whose onset (*t* = 0 ms) marks the point from which each ERP’s latency is measured*.

### Language-Related Event-Related Potentials

Typically, language processing experiments expose participants to semantically or syntactically violated (“critical”) stimuli (e.g., *“the blouse was on ironed.”), which is compared against unviolated (“control”) stimuli (“the blouse was ironed.”). Syntactic/morphosyntactic violations elicit the P600, a long-lasting positive deflection of voltage that peaks over centro-parietal areas of the brain around 600 ms post-stimulus (Osterhout and Holcomb, 1992; Kutas et al., 2006; Brouwer and Crocker, 2017). The P600 is often interpreted as an index of structure-related difficulties or reanalysis (Kutas et al., 2006, 693). It therefore stands to reason that the same P600 will be elicited by non-linguistic stimuli of languagelike systems.

Similarly, the N400—a centro-posterior negativity at 400 ms post-stimulus—and the LAN—a left anterior negativity, which also peaks around 400 ms post-stimulus—are of linguistic interest. The N400 is elicited by semantic anomalies (e.g., “the cat will bake.”) (Federmeier et al., 2002; Osterhout et al., 2004), and is interpreted as reflecting semantic integration in a combinatorial process, evidenced by a correlation between N400 amplitude and degree of semantic incongruence (Lau et al., 2008). The LAN often precedes the P600 elicited by morphosyntactic violations (e.g., *“the clerk were severely underpaid.”) (Barber and Carreiras, 2005; Molinaro et al., 2011; see also Friederici, 2002 for review).

### Arithmetic Sequence Violation Event-Related Potentials

Turning to recursive arithmetic sequences, consider an experiment where numbers in a series are presented one at a time. If the generating formula is simple, participants will deduce the formula and predict subsequent numbers. Then, MP predicts that a violation in this sequence would elicit some combination of the LAN, N400, and P600. This was indeed shown to be the case. While recording EEGs, experimenters showed participants seven numbers in sequence, each computed by the simple recursive formula *x_i_*_+1_ = *x_i_* + *c*, where *c* took the value ±2, 3, or 4 (e.g., “7 10 13 16 19 22 25”) and the final number was either correct (“...19 22 25”), widely incorrect (“...19 22 50”), or narrowly incorrect (“...19 22 24”) (Núñez-Peña and Honrubia-Serrano, 2004, pp. 132–133). The results showed an early left anterior negativity peaking around 250–300 ms (LAN), and a centro-parietal positivity peaking around 500–600 ms (P600). Furthermore, the P600 amplitude increased with widely incorrect endings, compared to narrowly incorrect endings (Núñez-Peña and Honrubia-Serrano, 2004, pp. 134–138).

For comparison, single arithmetic operation violations (e.g., “7 × 4…” “24”) are shown to elicit an N400 and a P3 (see section “Making Sense of the P3”) (Niedeggen et al., 1999, pp. 311–312). Structural differences may explain the N400. A single arithmetic operation corresponds to a single instance of Merge, while arithmetic sequences require a greater maximum depth of Merged subtrees (Degree of Merger) and employ an additional operation, Search (Ohta et al., 2013, p. 2), which refers back to previous elements in the hierarchical tree. This Search operation could explain the elicitation of the P600 by sequence violations and its absence by single operation violations, although further research is required to test this hypothesis.

### Musical Prolongation Violation Event-Related Potentials

Recall that GTTM’s PRWFR 1 states that there exists a single prolongational head, which governs all subordinate pitch events. In contradiction to this rule, a prolongation violation is a pitch event that disagrees with its prolongational head (i.e., an out-of-key note/chord). Such a pitch event would cause a breakdown and/or reanalysis of the prolongational hierarchy, and by MP prediction should elicit a languagelike neural response.

As with the arithmetic sequence, this turns out to be the case. Patel et al. played musical phrases to musically trained participants while recording EEG data (Patel et al., 1998, p. 718). Each phrase consisted of block chords in an established key, at the end of which a target chord was presented as an in-key chord (control), a nearby-key chord, or a distant-key chord (Patel et al., 1998, p. 722). EEG results showed a late, centro-parietal positivity peaking at 600 ms post-stimulus (i.e., P600) (Patel et al., 1998, p. 723). Moreover, the strongly violated distant-key condition elicited a greater P600 amplitude compared with the weakly violated nearby-key condition (Patel et al., 1998, p. 724). Finally, an anterior negativity was found 300–400 ms post-stimulus, though in contrast to the LAN, its distribution was right-lateralized and maximized over anterior-temporal areas (termed RATN) (Patel et al., 1998, p. 726). These results agree with other musical ERP studies (see, e.g., Besson and Faïta, 1995) and with the arithmetic sequence violation ERP whose amplitude was also modulated by degree of violation.

Jackendoff claims that in music, meter is the most consistent with language in terms of hierarchical structure (Jackendoff, 2009, p. 203). Thus, we might expect metric violations to elicit the P600. However, ERP studies on metric deviance report the mismatch negativity (MMN)—a fronto-central negativity peaking around 150–250 ms that is sensitive to infrequent change in repetitive auditory sequences—and a P3 (see section “Making Sense of the P3”) (see James et al., 2012, pp. 2762–2765; Bouwer et al., 2014, pp. 5–8). Here, experimental design dictated that deviant stimuli were constructed by omitting beats or by changing the accent pattern (Bouwer et al., 2014, p. 2). With constant tempo and time signature, such deviations may be interpreted as metric elaborations (analogous to prolongational elaborations) rather than metric violations, thus eliciting the simpler MMN. In other words, metric elaboration may not cause listeners to reanalyze the underlying hierarchical structure.

The results of the arithmetic sequence violation and musical prolongation violation studies show that these violations are, in essence, processed as or very similarly to morphosyntactic violations and that the LAN and P600 are not language-specific, but may instead have languagelike-specificity.

## Making Sense of the P3

The P3 is a centro-parietal positivity around 300 ms, elicited by related but improbable or infrequent events and consists of two subcomponents, P3a and P3b. P3a is an earlier component with a central maximum, related to attentional mechanisms, while P3b is a later component with a parietal maximum, related to attention and memory processing, and modulated by difficulty (Polich, 2007, pp. 2128–2135).

Bouwer et al.’s metric violation elicited a P3a (Bouwer et al., 2014, p. 4), which is consistent with the interpretation that the metric violation requires attention but no deeper reanalysis of the underlying hierarchical structure.

On the other hand, some researchers propose that the P600 belongs to the P3 “family,” evidenced by the observation that both P3b and P600 amplitudes are modulated by difficulty and latencies are modulated by reaction time (Sassenhagen et al., 2014, pp. 32–33). Under this interpretation, MP would predict that any P3b-eliciting stimulus is languagelike, or else the P3b/P600 cannot be languagelike-specific. Consider one experiment where viewing a video of a man attempting to cut bread with an iron was shown to elicit (a rather late) P3b (as well as an N400) (Sitnikova et al., 2008, pp. 2047–2054). If the P3b is languagelike-specific, that implies that this and similar stimuli are processed in a recursive, hierarchical languagelike way (e.g., through combinatorial processes of semantic information contained in the video). It is easy to extend this to the argument that nearly all complex systems are hierarchical in nature. In fact, Pinker and Jackendoff argue that the problem is not that too few systems are languagelike, but rather too many are (Pinker and Jackendoff, 2005, p. 230). This does not render FL meaningless, but rather demonstrates how it uniquely equips us to approach many different complex systems in a way that organisms without FL cannot.

## Conclusions

In this mini-review, I have treated “languagelike” as a technical term defined by recursion criteria. I have shown that ERP studies demonstrate cognitive overlap (LAN, N400, and P600) between language and subdomains of math, and music, supporting MP prediction that cognitive processing of different languagelike systems is qualitatively distinct. I have also suggested that since some languagelike systems do not elicit languagelike ERPs, if MP prediction is true, then these discrepancies must be accounted for, for example, by structural inconsistencies or by reinterpreting the P600 as belonging to the P3 family.

It is outside the scope of this mini-review to dissociate languagelike ERPs from general cognitive function, and until such dissociation is made, languagelike-specificity cannot be indisputably confirmed. To that end, it is important that future research explore this and similar issues by framing hypotheses in light of current linguistic theory. It is also important that greater efforts be made for cross-communication between linguistic and non-linguistic neuroscientific areas of research.

Regardless, it is clear that the data considered here are compatible with the interpretation that the LAN, N400, and/or P600 have languagelike-specificity and that their elicitation from different languagelike systems indicates a qualitatively distinct processing mechanism for languagelike systems, as predicted by MP.

## Author Contributions

The author confirms being the sole contributor of this work and has approved it for publication.

### Conflict of Interest

The author declares that the research was conducted in the absence of any commercial or financial relationships that could be construed as a potential conflict of interest.

## Footnotes

^1^The astute reader may notice that the claim predates MP. This does not, however, lessen its pertinence to MP, which is “simply a continuation of what has been undertaken from the earliest years [of generative grammar]” (Chomsky, 2011, p. 263). The concept is salient throughout MP literature, but wording tends to be less succinct (for purposes here), and more evolution-centric than predictive (see Pinker and Jackendoff, 2005, p. 229; Ott, 2007, p. 7; etc.).

^2^Precisely, it is FL in the narrow sense (FLN) that is characterized by recursion. This is described in contrast to FL in the broad sense (FLB), which includes interface systems (Hauser et al., 2002, p. 1569). The distinction is important but not relevant here, hence the simplified use of FL instead of FLN.

^3^GTTM defines direct and recursive elaboration such that event *e_i_* is a *direct elaboration* of *e_j_* if *e_i_*’s hierarchical branch terminates on *e_j_*’s branch; and *e_i_* is a *recursive elaboration* of *e_j_* if it is a direct elaboration of *e_i_* or if a series of direct elaborations lead to *e_i_*’s branch (Lerdahl and Jackendoff, 1983, p. 214).

## References

[ref1] Barber H. Carreiras M. (2005). Grammatical gender and number agreement in Spanish ERP: an ERP comparison. J. Cogn. Neurosci. 17, 137–153. 10.1162/0898929052880101, PMID: 15701245

[ref2] Besson M. Faïta F. (1995). An event-related potential (ERP) study of musical expectancy: comparison of musicians with nonmusicians. J. Exp. Psychol. Hum. Percept. Perform. 21, 1278–1296. 10.1037/0096-1523.21.6.1278

[ref3] Bouwer F. L. Van Zuijen T. L. Honing H. (2014). Beat processing is pre-attentive for metrically simple rhythms with clear accents: an ERP study. PLoS One 9, 1–9. 10.1371/journal.pone.0097467 PMC4037171 24870123

[ref4] Brouwer H. Crocker M. W. (2017). On the proper treatment of the N400 and P600 in language comprehension. Front. Psychol. 8, 1–5. 10.3389/fpsyg.2017.01327 28824506 PMC5539129

[ref5] Burle B. Spieser L. Roger C. Casini L. Hasbroucq T. Vidal F. (2015). Spatial and temporal resolutions of EEG: is it really black and white? A scalp current density view. Int. J. Psychophysiol. 97, 210–220. 10.1016/j.ijpsycho.2015.05.004, PMID: 25979156 PMC4548479

[ref6] Chomsky N. (1965). Aspects of the theory of syntax. Cambridge, Massachusetts: The MIT Press.

[ref7] Chomsky N. (1995). The minimalist program. 20th Anniversary. Cambridge, Massachusetts: The MIT Press.

[ref8] Chomsky N. (2011). Language and other cognitive systems. What is special about language? Lang. Learn. Dev. 7, 263–278. 10.1080/15475441.2011.584041

[ref9] Chomsky N. Gallego Á. J. Ott D. (2017). Generative grammar and the faculty of language: insights, questions, and challenges. Cat. J. Linguist. 1–38. Available at: https://ling.auf.net/lingbuzz/003507

[ref10] Everett D. L. (2005). Cultural constraints on grammar and cognition in Pirahã another look at the design features of human language. Sprachwissenschaft 46, 621–646. 10.1515/9783110588972-072

[ref11] Federmeier K. Mclennan D. de Ochoa E. Marta K. (2002). The impact of semantic memory organization and sentence context information on spoken language processing by younger and older adults: an ERP study. Psychophysiology 39, 133–146. 10.1111/1469-8986.3920133, PMID: 12212662

[ref12] Friederici A. D. (2002). Towards a neural basis of auditory sentence processing. Trends Cogn. Sci. 6, 78–84. 10.1016/S1364-6613(00)01839-8, PMID: 15866191

[ref13] Hauser M. D. Chomsky N. Tecumseh Fitch W. (2002). The faculty of language: what is it, who has it, and how did it evolve? Science 298, 14–42. 10.1017/CBO9780511817755.002 12446899

[ref14] Jackendoff R. (2009). Parallels and nonparallels between language and music. Music. Percept. 26, 195–204. 10.1525/mp.2009.26.3.195

[ref15] James C. E. Michel C. M. Britz J. Vuilleumier P. Hauert C.-A. (2012). Rhythm evokes action: early processing of metric deviances in expressive music by experts and laymen revealed by ERP source imaging. Hum. Brain Mapp. 33, 2751–2767. 10.1002/hbm.21397, PMID: 21932257 PMC6870197

[ref16] Kutas M. Cyma K. Petten V. Kluender R. (2006). “Psycholinguistics electrified II (1994–2005)” in Handbook of psycholinguistics. 2nd Edn. eds. Traxler M. J. Gernsbacher M. A. (London, UK: Academic Press), 659–724.

[ref17] Kutas M. Federmeier K. D. (2011). Thirty years and counting: finding meaning in the N400 component of the event-related brain potential (ERP). Annu. Rev. Psychol. 62, 621–647. 10.1146/annurev.psych.093008.131123, PMID: 20809790 PMC4052444

[ref18] Lau E. F. Phillips C. Poeppel D. (2008). A cortical network for semantics: (De)constructing the N400. Nat. Rev. Neurosci. 9, 920–933. 10.1038/nrn2532, PMID: 19020511

[ref19] Lerdahl F. Jackendoff R. (1983). A generative theory of tonal music. Cambridge, Massachusetts, USA: The MIT Press.

[ref20] Molinaro N. Barber H. A. Carreiras M. (2011). Grammatical agreement processing in reading: ERP findings and future directions. Cortex 47, 908–930. 10.1016/j.cortex.2011.02.019, PMID: 21458791

[ref21] Nevins A. Pesetsky D. Rodrigues C. (2009). Piraha exceptionality: a reassessment. Language 85, 355–404. 10.1353/lan.0.0107

[ref22] Niedeggen M. Rösler F. Jost K. (1999). Processing of incongruous mental calculation problems: evidence for an arithmetic N400 effect. Psychophysiology 36, 307–324. 10.1017/S0048577299980149, PMID: 10352554

[ref23] Nieuwland M. S. (2019). Do ‘early’ brain responses reveal word form prediction during language comprehension? A critical review. Neurosci. Biobehav. Rev. 96, 367–400. 10.1016/j.neubiorev.2018.11.019, PMID: 30621862

[ref24] Núñez-Peña M. I. Honrubia-Serrano M. L. (2004). P600 related to rule violation in an arithmetic task. Cogn. Brain Res. 18, 130–141. 10.1016/j.cogbrainres.2003.09.010, PMID: 14736572

[ref25] Ohta S. Fukui N. Sakai K. L. (2013). Syntactic computation in the human brain: the degree of merger as a key factor. PLoS One 8, 1–16. 10.1371/journal.pone.0056230, PMID: 23437097 PMC3577822

[ref26] Osterhout L. Holcomb P. J. (1992). Event-related brain potentials elicited by syntactic anomaly. J. Mem. Lang. 806, 785–806. 10.1016/0749-596X(92)90039-Z

[ref27] Osterhout L. McLaughlin J. Kim A. Greenwald R. Inoue K. (2004). “Sentences in the brain: event-related potentials as real-time reflections of sentence comprehension and language learning” in The on-line study of sentence comprehension: Eyetracking, ERP, and beyond. eds. Carreiras M. Clifton Jr. C. (New York: Psychology Press, New York and Hove).

[ref28] Ott D. (2007). “Reverse-engineering the language faculty: origins and implications of the minimalist program” in Harvard Working Papers in Linguistics. Vol. 12, 77–90.

[ref29] Patel A. D. Gibson E. Ratner J. Besson M. Holcomb P. J. (1998). Processing syntactic relations in language and music: an event-related potential study. J. Cogn. Neurosci. 10, 717–733. 10.1162/089892998563121, PMID: 9831740

[ref30] Pinker S. Jackendoff R. (2005). The faculty of language: what’s special about it? Cognition 95, 201–236. 10.1016/j.cognition.2004.08.004, PMID: 15694646

[ref31] Polich J. (2007). Updating P300: an integrative theory of P3a and P3b. Clin. Neurophysiol. 118, 2128–2148. 10.1016/j.clinph.2007.04.019, PMID: 17573239 PMC2715154

[ref32] Sassenhagen J. Schlesewsky M. Bornkessel-Schlesewsky I. (2014). The P600-as-P3 hypothesis revisited: single-trial analyses reveal that the late EEG positivity following linguistically deviant material is reaction time aligned. Brain Lang. 137, 29–39. 10.1016/j.bandl.2014.07.010, PMID: 25151545

[ref33] Sitnikova T. Holcomb P. J. Kiyonaga K. A. Kuperberg G. R. (2008). Two neurocognitive mechanisms of semantic integration during the comprehension of visual real-world events. J. Cogn. Neurosci. 20, 2037–2057. 10.1162/jocn.2008.20143, PMID: 18416681 PMC2673092

[ref34] Stemmer B. Rodden F. A. (2015). “Functional brain imaging of language processes” in International encyclopedia of the social & behavioral sciences. 2nd Edn. Vol. 9, ed. Wright J. D. (London, UK: Elsevier).

[ref35] Watumull J. Hauser M. D. Roberts I. G. Hornstein N. (2014). On recursion. Front. Psychol. 4, 1–7. 10.3389/fpsyg.2013.01017 PMC3884515 24409164

